# Genome Sequence of a Leptospira montravelensis Strain Isolated from Donkey Urine during a Leptospirosis Outbreak in Sardinia Island, Italy

**DOI:** 10.1128/mra.01009-22

**Published:** 2023-01-04

**Authors:** Ivana Piredda, Loris Bertoldi, Valentina Chisu

**Affiliations:** a Istituto Zooprofilattico Sperimentale of Sardinia, Animal Health Department, Sassari, Italy; b BMR Genomics srl, Padua, Italy; Indiana University, Bloomington

## Abstract

Leptospirosis is a neglected zoonosis caused by pathogenic spirochetes classified within the genus *Leptospira*. Among saprophytic species, Leptospira montravelensis was previously reported only from environmental samples. Here, we report the whole-genome sequence and annotation of a strain of Leptospira montravelensis that was isolated from donkey urine during a leptospirosis outbreak in Sardinia, Italy.

## ANNOUNCEMENT

Leptospirosis is a zoonotic disease caused predominantly by pathogenic *Leptospira* species (1). The genus *Leptospira* has been recently reorganized into two clades (P and S) and four subclades (P1, P2, S1, and S2) (2). Leptospira montravelensis has been classified within subclade S1 (among the saprophytic species). Here, we report the whole-genome sequence of a *Leptospira* strain that was isolated from a sample of urine collected from one donkey in a farm in Sardinia. The urine was cultured in semisolid medium using Ellinghausen-McCullough-Johnson-Harris (EMJH)-enriched commercial medium (Difco, BD, Franklin Lakes, NJ, USA) supplemented with 5-fluorouracil (5-FU) (2 g/L), incubated at 30°C, and observed weekly under a dark-field microscope. After isolation of the bacterium, a small drop of the culture was transferred to a solid EMJH agar plate. When the density reached 1 × 10^8^ leptospires, genomic DNA (gDNA) was extracted with the DNeasy blood and tissue kit (Qiagen, Hilden, Germany).

An Illumina library was constructed with the Celero PCR workflow with enzymatic fragmentation kit, checked with a BioAnalyzer (Agilent, Santa Clara, CA, USA), and quantified with a Qubit fluorometer (Thermo Fisher Scientific, Bedford, MA, USA). The library was sequenced according to the v3 paired-end 300-bp read strategy, which yielded 1,366,977 paired-end sequences. Read preprocessing was performed first with FastQC v0.11.9 and then with Fastp v0.23.2, in order to remove short (lengths of <150 bp), low-quality (Q scores of <25), and low-complexity (30%) reads. Clean reads were then profiled with MetaPhlAn v3.0.14 with the ChocoPhlAn database (release mpa_v30_CHOCOPhlAn_201901), excluding possible contamination due to other bacterial species. Finally, reads were assembled with SPAdes v3.14.5 with standard parameters, which produced 32 contigs of >500 bp. Assembly metrics were calculated with QUAST v5.0.2. The genome size was 4,173,219 bp, with an average coverage depth of 183×, an *L*_50_ value of 1, an *N*_50_ value of 2,971,169 bp, and an estimated GC content of 37.37%. Genome completeness was estimated using benchmarking universal single-copy orthologs (BUSCO) v5.3.0 with the spirochaetia_odb10 database, which yielded good results (235 complete and single-copy BUSCOs, 3 fragmented BUSCOs, and just 1 missing BUSCO). Furthermore, gene prediction and annotation were performed with Prokka v1.14.6 and eggNOG-mapper v2.1.7 (3), respectively, which yielded 3,945 possible open reading frames (3,909 coding sequences [CDSs], 35 tRNAs, and 1 transfer-messenger RNA). An automatic annotation was also added by the NCBI Prokaryotic Genome Annotation Pipeline (PGAP), which yielded 3,939 genes (3,895 CDSs, 7 rRNAs, 35 tRNAs, and 2 noncoding RNAs). In addition, the average nucleotide similarity (4) between our isolated strain and a L. montravelensis strain (GenBank accession number NZ_RQFN00000000) was 99.30%.

### Data availability.

This whole-genome shotgun project has been deposited in DDBJ/ENA/GenBank under the accession number JANLJQ000000000 (BioProject accession number PRJNA867047, BioSample accession number SAMN30183847, and SRA accession number SRR22308640). The version described in this paper is version JAHZNM010000000.
